# Atypical Nodular Hidradenoma Versus Low-Grade Hidradenocarcinoma in a Young Female Patient: A Case Report and Review of Literature

**DOI:** 10.7759/cureus.32772

**Published:** 2022-12-21

**Authors:** Alexander M Knops, Kathleen E McClain, Nicole L Aaronson

**Affiliations:** 1 Department of Otolaryngology, Thomas Jefferson University Hospital, Philadelphia, USA; 2 Department of Otolaryngology, Dell Children's Medical Center, Austin, USA; 3 Department of Otolaryngology and Pediatrics, Thomas Jefferson University Sidney Kimmel School of Medicine, Philadelphia, USA; 4 Department of Surgery, Section of Otolaryngology, Nemours Children's Health, Delaware Valley, Wilmington, USA

**Keywords:** atypical nodular hidradenoma, postauricular mass, hidradenocarcinoma, pediatric otolaryngology, hidradenoma

## Abstract

The objective of this study was to discuss an unusual postauricular mass in a pediatric patient. This mass had a broad differential including congenital anomaly, neoplasm, infection, and lymphovascular malformation. Atypical nodular hidradenoma is a rare adnexal tumor that is difficult to differentiate from hidradenocarcinoma. It is a rare entity, but especially rare in the pediatric population. This study aims to provide guidance on diagnosing hidradenoma and distinguishing it from hidradenocarcinoma through case presentation with a review of the literature. The patient in this report underwent wide location resection with close surveillance and has been disease-free during follow-up.

## Introduction

Nodular hidradenomas are rare skin tumors of sweat gland origin, typically presenting as slow-growing nodules on the head, anterior trunk, or upper limbs. However, the clinical presentation can be variable and is often not sufficient for diagnosis [1]. Differential diagnosis must include sebaceous carcinoma, another slow-growing cutaneous neoplasm [2]. In nodular hidradenomas, patients between the ages of 40 and 80 years are the most commonly affected demographic [3], whereas sebaceous carcinoma is more common in individuals older than 70 years. Presentations in children are exceedingly rare [4]. Limited clinical information exists on these tumors and their management, particularly in the pediatric population. We present the youngest reported case of atypical hidradenoma versus low-grade hidradenocarcinoma and a review of the literature.

## Case presentation

**Figure 1:**
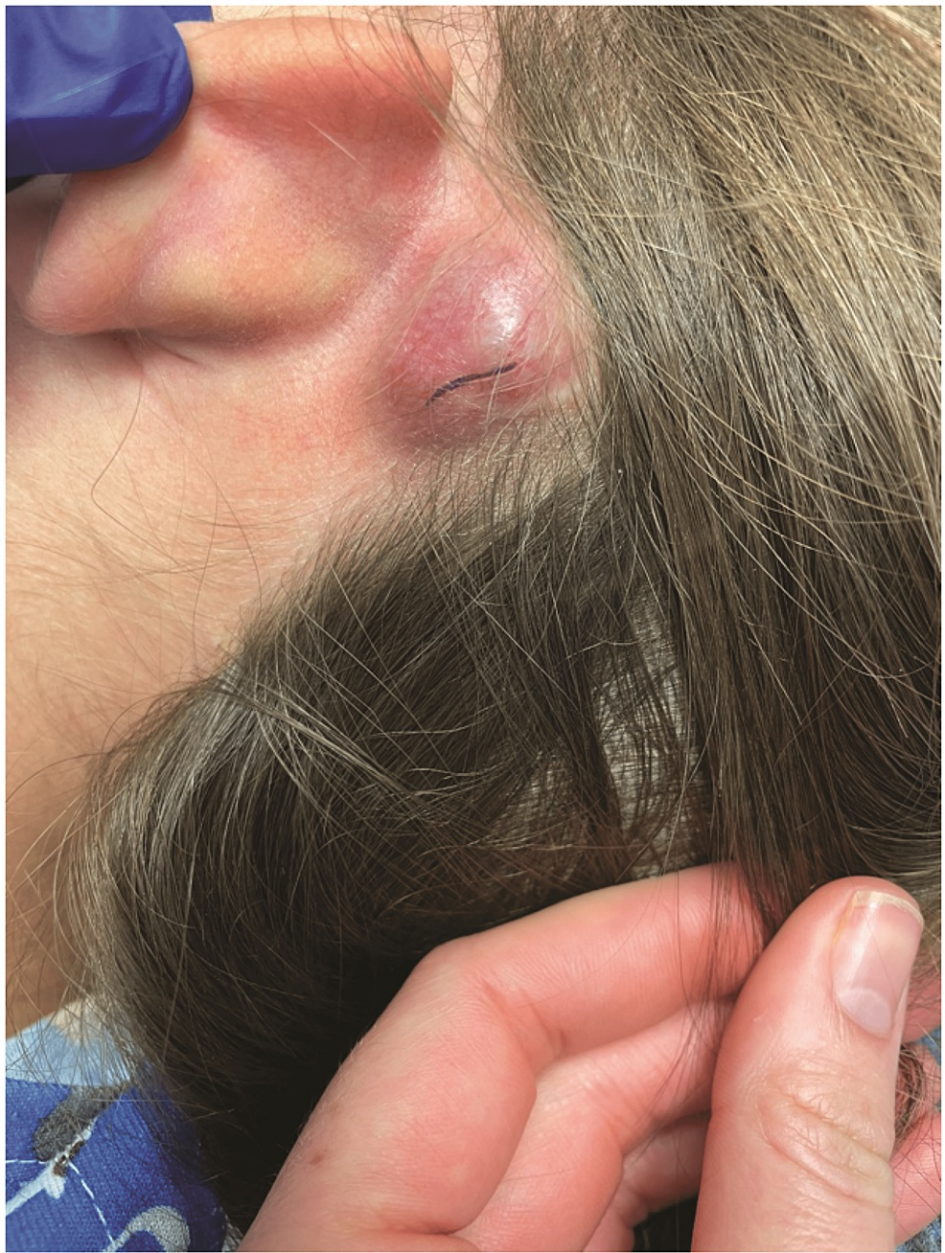
Lesion Preoperative examination showed a 2 cm firm, non-fluctuant, postauricular mass at superior aspect of the auricle with thinning and violaceous discoloration of the overlying skin.

An eight-year-old female presented to the clinic after discovering a post-auricular nodule approximately 10 days prior. No systemic symptoms were noted. The lesion was 2.0 x 1.7 x 0.7 cm, soft to palpation, well-circumscribed, and red-purple in color with no drainage or ulceration (Figure 1). Ultrasound had been conducted at an outside facility and demonstrated vascular flow through the lesion. Testing for infectious causes including Lyme, Bartonella, and Epstein-Barr virus was all negative. Complete blood count was within normal limits with no elevation of white blood cell counts. Given the assumption of a benign diagnosis, surgical excision was performed without biopsy. Intraoperatively, the lesion was very adherent to the overlying skin. The underlying temporal bone was noted to be scalloped and irregular.

**Figure 2:**
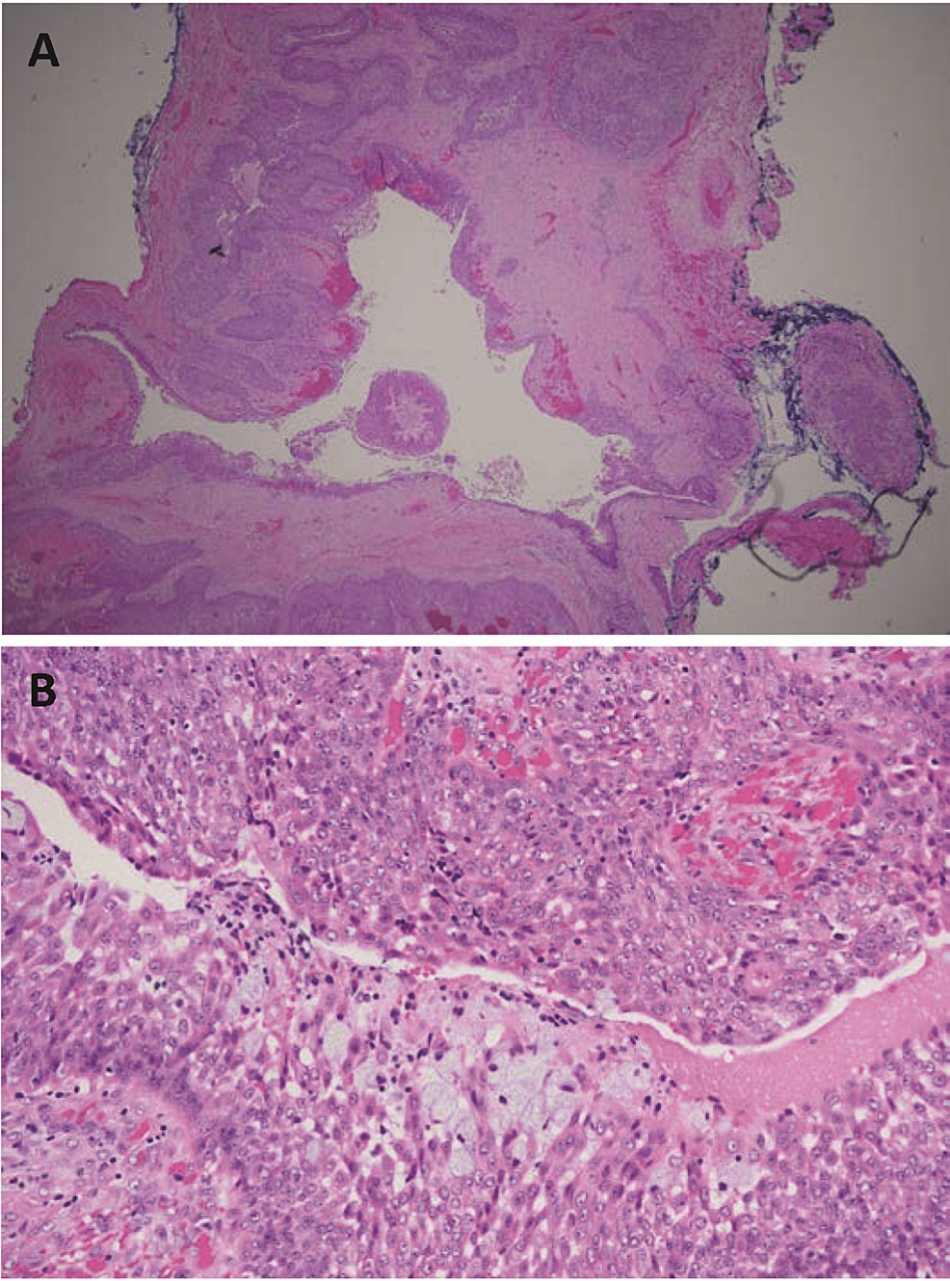
Histological appearance of the lesion Histological appearance of the lesion under hematoxylin-and-eosin staining. (A) cystic tumor with deep invaginations at 40X magnification; (B) 400X magnified section showing squamoid, clear cell, and mucinous differentiation with hyaline stroma and focal necrosis.

Pathologic evaluation of the excised lesion demonstrated squamoid, clear cell, and mucinous differentiation, characteristic of nodular hidradenoma. Areas of necrosis were also noted within the specimen, leading to an inability to rule out low-grade hidradenocarcinoma (Figure 2). Other pathologic features of malignancy including hyperchromatism, nuclear pleomorphism, and perineural or lymphovascular invasion were not present. Gene fusion sequencing demonstrated a *CRTC1-MAML2* fusion.

Given the ambiguity of the specimen and after discussion with the family, a decision was made to undergo a more thorough malignancy work-up and wide local excision to ensure clear margins. Positron emission tomography/magnetic resonance imaging did not demonstrate any areas of concern for metastasis and the patient underwent re-excision of the tumor with a fasciocutaneous advancement flap for closure of the 5 x 8 cm defect. The family elected for genetic testing, which showed no variants in first-tier cancer markers including a variant in the *PTEN*, *FLCN*, or mismatch repair genes (*MLH1*, *MSH2*, *MSH6*, and *PMS2*). The patient has done well in the postoperative period and will be surveilled closely for local or distant recurrence with serial magnetic resonance images every three months and alternating chest radiographs and chest computed tomography scans for the first year.

## Discussion

Hidradenocarcinoma is a rare tumor that was first identified in 1954. It typically arises de novo without any family history or underlying genetic syndrome that is noted. Incidence increases with age [5]. While there are multiple case reports in the literature, there are none in a pediatric patient. Evaluation of the Surveillance, Epidemiology and End Results (SEER) database has shown the head and neck to be the primary site for these tumors [6]. The youngest patient with hidradenocarcinoma in this database was 44 years old with a median age of diagnosis of 58%. There was a slight male predominance (58%), and the majority of patients (70%) were of white non-Hispanic origin [7].

Differentiation between hidradenoma and hidradenocarcinoma is difficult, even with histologic evaluation. Among skin adnexal tumors, sweat gland carcinoma, such as hidradenocarcinoma, was most prone to diagnostic discrepancies. Up to 1.8% of patients reviewed had subtype misclassifications with a predicted clinical impact [8]. Hidradenocarcinomas are a rare diagnosis, with an estimated incidence of <0.05% [9]. Hidradenocarcinoma typically includes pathologic features such as necrosis, infiltrative growth patterns, deep extension, nuclear pleomorphism, invasion of vascular or neural structures, or increased mitoses. However, many hidradenocarcinomas do not possess all of the criteria listed. Atypical nodular hidradenomas possess some of the worrisome features seen in hidradenocarcinoma and have been linked with increased rates of recurrence [10]. Malignant transformation of hidradenoma to hidradenocarcinoma has been described, both in untreated and inadequately excised lesions [11]. Hidradenocarcinomas have an aggressive clinical course, often characterized by systemic metastasis and local recurrence. These tumors metastasize most commonly to the bone, lymph nodes, or visceral organs [12].

Reliable diagnostic markers for hidradenoma have yet to be established. Tumors are usually diagnosed on the basis of hematoxylin-eosin staining, although immunohistochemical staining for anion exchanger (AE) AE1/AE3, epithelial membrane antigen, and carcinoembryonic antigen can be of assistance. A t(11;19) translocation of *METC1*/*MAML2 *has been reported in approximately 50% of cutaneous hidradenomas [13,14]. The resulting oncogenic fusion results in activation of *GREB*-dependent transcription. The *METC1*/*MAML2 *fusion has been identified in other glandular tumor types including mucoepidermoid carcinoma and Warthin’s tumors [15,16]. Paradoxically, this fusion is less common in hidradenocarcinoma and its absence in the remaining 50% of hidradenomas limits its diagnostic utility.

Wide local excision, with histological confirmation of clear margins, remains the best modality for the management and reduction of recurrence or transformation of hidradenoma. There is no consensus regarding optimal margins. Local recurrence is common after wide local excision of nodular hidradenoma, up to 10% [16].

Wide local excision is also the mainstay of treatment for hidradenocarcinoma. Sentinel lymph node biopsy may detect subclinical metastases and can be considered [17]. In the absence of known metastasis, the role of selective neck dissection is under debate--currently, no clear evidence exists to suggest its necessity [12]. The post-surgical recurrence rate of hidradenocarcinoma is 10%-50% [12,18]. Given the aggressive course of hidradenocarcinoma, radiotherapy has been suggested for adjuvant treatment, but the low incidence has limited the feasibility of clinical trials [19]. House et al. suggest that Mohs micrographic surgery may be beneficial for oncologic excision, particularly for recurrent or large lesions [20].

## Conclusions

Atypical nodular hidradenoma is a rare adnexal tumor that is difficult to differentiate from hidradenocarcinoma. High suspicion for this tumor and engagement of a pathology team with expertise in adrenal tumors is key for diagnosis. Wide local excision is the mainstay of treatment for both atypical nodular hidradenoma and hidradenocarcinoma. If suspicion of either pathology is present, obtaining surgical margins at the time of initial surgery resection is reasonable. Given the high rate of local recurrence, close follow-up is recommended. Our patient was followed with imaging every three months for the first year. With no evidence of recurrence after one year, imaging studies were spaced out to six-month intervals.
